# Venous Thromboembolism Risk Assessment in Prolonged Fasting During Ramadan: The Global Ramadan Free Thrombosis Project

**DOI:** 10.7759/cureus.89406

**Published:** 2025-08-05

**Authors:** Joseph Caprini, Ayman Fakhry, Sohiel Nagib, Abdullah H Al-Mallah, Eslam M Barsim, Mohamed Farrag, Ahmed Abouelseoud, Omar Alnadi, Mahmoud Moner, Ziad M Ghazy, Rahma A Seken, Muhammad Jabr, Malika Boucelma, Ayman Zyada

**Affiliations:** 1 Vascular Surgery, Pritzker School of Medicine at the University of Chicago, Chicago, USA; 2 Vascular Surgery, Egyptian Military Academy, Cairo, EGY; 3 Vascular Surgery, Royal Vascular Center, Alexandria, EGY; 4 Medical Education, Maastricht University, Maastricht, NLD; 5 Medical Education, Al-Azhar University, Cairo, EGY; 6 Vascular Surgery, Al-Azhar University, Cairo, EGY; 7 Vascular Surgery, Faculty of Medicine, Menofia University, Menofia, EGY; 8 Surgery, Alexandria Main University Hospital, Alexandria, EGY; 9 Vascular Surgery, Alexandria Main University Hospital, Alexandria, EGY; 10 General Surgery, Abu Qir General Hospital, Alexandria, EGY; 11 Surgery and Medicine, Al-Ahrar Teaching Hospital, Al-Sharqia, EGY; 12 Vascular Surgery, Abu Qir General Hospital, Alexandria, EGY; 13 Faculty of Medicine, Al-Azhar University, Damietta, EGY; 14 Medicine and Surgery, Abu Qir General Hospital, Alexandria, EGY; 15 Internal Medicine, Bachir Mentouri Hospital, Kouba, DZA; 16 Vascular Surgery, University Hospitals of Leicester NHS Trust, Leicester, GBR

**Keywords:** caprini score, deep venous thrombosis (dvt), fasting ramadan, risk assessment model, venous thromboembolism, vte prophylaxis

## Abstract

Background

Fasting during the month of Ramadan is practiced by over a billion Muslims worldwide. This religious observance, which involves complete abstention from food and fluids during daylight hours, may contribute to dehydration and increase the risk of venous thromboembolism (VTE), particularly in hot climates. Despite this theoretical concern, limited clinical evidence exists on the actual incidence and risk of VTE associated with prolonged fasting. This study aimed to assess the risk of VTE in fasting individuals during Ramadan using the Caprini risk assessment model and propose a prophylactic strategy tailored for the fasting population.

Methodology

A multicenter, observational study was conducted across several countries during the Ramadan period. Standard Caprini scores were calculated, and additional fasting-specific risk factors, including fasting duration, temperature exposure, physical activity, and hydration, were recorded. The presence of varicose veins and a history of thrombophilia were also evaluated. VTE events were tracked and analyzed during the fasting period.

Results

A proportion of participants developed VTE during the fasting period. Those who experienced thrombotic events had higher Caprini risk scores and a greater prevalence of contributing factors such as varicose veins, venous reflux, and thrombophilia. All events occurred during the fasting period and were not attributed to other medical conditions.

Conclusions

Ramadan fasting may unmask or exacerbate thrombotic risk, particularly in individuals with pre-existing venous disease or thrombophilia. Risk stratification using the Caprini score, supplemented with fasting-specific factors, can help identify at-risk individuals. Targeted preventive strategies, including patient education, fluid management, and medical prophylaxis for high-risk groups, should be considered to ensure safe fasting practices during Ramadan.

## Introduction

Fasting during Ramadan is observed by most of the 1.8 billion Muslims around the world. It lasts for one month, according to the lunar calendar year, and involves abstaining from all food and drink from dawn to sunset [1]. Theoretically, fasting can induce dehydration, which may act as a triggering factor for venous thrombosis, together with other contributing factors; however, clinically diagnosed venous thromboembolism (VTE) after prolonged fasting has been scarcely reported in the literature [2]. Studies examining the risk of Ramadan fasting in patients with VTE are lacking. Ramadan fasting in pregnant patients with cardiovascular conditions has been discussed [3]. In the absence of any studies assessing the above conditions in Ramadan fasting, we advise that any disease where there may be a significant risk of dehydration or hypotension should be classed as at least *high risk* [4]. Significant dehydration in this otherwise healthy and active population, defined as having no chronic illnesses, not on anticoagulants, and normally mobile, most likely contributes to a transient state of hypercoagulability. It is unknown if an undiagnosed underlying predisposition to thrombosis, such as a hereditary thrombophilia, plays a role or if fasting can affect coagulability in another manner [5]. Fasting-related physiologic changes have been hypothesized to influence renal fluid and electrolyte handling, potentially leading to dehydration in certain individuals. One proposed mechanism involves decreased glucose intake during fasting, which may reduce sodium reabsorption in renal proximal tubules by impairing glucose-sodium cotransport. This could theoretically contribute to natriuresis and osmotic diuresis, although this mechanism has not been directly validated in fasting populations [6]. This state likely results in hypercoagulability induced by hemoconcentration and hyperviscosity, as reflected in part by increased biochemical parameters such as hematocrit, plasma proteins, plasma, and urine osmolality [7]. Other conditions in which VTE likely occurs because of dehydration have been described, such as following ischemic strokes, intense exercises, and gastroenteritis [7]. According to some studies, seasonal variation of VTE incidence could hypothetically be related to dehydration, occurring more often at higher temperatures [5]. In this study, we aimed to conduct a risk assessment for VTE among fasting Muslims during Ramadan and suggest a prophylaxis protocol against VTE in Ramadan with the potential impact of fasting-specific factors.

## Materials and methods

This multicenter, observational study was conducted during the 2025 Ramadan across seven countries in regions with typically warm climates, including Egypt, Algeria, Tunisia, Iraq, Lebanon, Syria, and Turkey. A total of 311 adult Muslim participants, aged 18 to 73 years, were enrolled. All participants observed full-day fasting during the full 30 days of Ramadan, abstaining from food and fluid from dawn to sunset each day, with a mean fasting duration of 14.02 ± 0.08 hours per day over 30 consecutive days.

VTE risk was assessed using the Caprini risk assessment model [8]. In addition to standard Caprini score components, we introduced the following supplementary fasting-specific risk factors: average daily fasting hours, ambient daytime temperatures, and self-reported physical activity duration. Ambient temperature data were collected using daily averages from local meteorological sources corresponding to each study site during Ramadan. These additional parameters were recorded to determine whether prolonged fasting in warm climates contributes to increased thrombotic risk. The additional risk components are summarized in Table 1.

**Table 1:** Additional risk components.

Points	One point	Two points	Three points	Four points
Fasting hours	<12 hours	12–15 hours	15–18 hours	>18 hours
Temperature	<30°C	30–32°C	32–34°C	>34°C
Thrombophilia history	Positive	Nil		
Limited mobility	Positive	Nil		

Based on the total risk score calculated using the additional risk components, participants were classified into four VTE risk categories. A score of 0 to 2 indicated low risk, while a score of 3 to 4 indicated moderate risk. Participants with scores ranging from 5 to 7 were considered at high risk, and those with scores between 8 and 10 were placed in the very high risk category.

Participants were evaluated clinically for the presence of varicose veins, and detailed venous assessments were performed using the proposed Varicose Vein Analysis Sheet. This included the side of involvement, clinical-etiological-anatomical-pathophysiological (CEAP) classification, history of previous venous treatment, and the presence of venous reflux on duplex examination (Table 2). This data allowed for correlation between superficial venous pathology and thrombotic events.

**Table 2:** Detection and analysis of varicose veins. CEAP = clinical-etiological-anatomical-pathophysiological

Assessment category	Variable/Description
Duration of varicose veins	Number of years since onset
Site of varicosity	Thigh, leg, foot
Side affected	Right, left, both
CEAP stage classification	From C1 to C6
Presence of vein reflux	Yes/No (duplex-confirmed)
Deep venous competency	Yes/No (based on duplex scan)
History of superficial venous thrombosis	Yes/No
Prior treatment	Yes/No (e.g., surgery, sclerotherapy)

No participant received pharmacologic thromboprophylaxis, as the study was non-interventional. The aim was to observe the natural incidence of VTE. Any VTE events diagnosed during Ramadan were recorded and confirmed through appropriate imaging modalities such as duplex ultrasonography or CT venography. These events included superficial thrombophlebitis, distal and proximal deep vein thrombosis (DVT), and iliofemoral thrombosis. All findings were stratified by Caprini score and analyzed to determine clinical significance.

Statistical analysis included comparisons between VTE and non-VTE groups using independent t-tests and chi-square analysis for continuous and categorical variables, respectively. A p-value <0.05 was considered statistically significant. Additional comparative data are presented to highlight the relationships between VTE events and various clinical and demographic parameters.

## Results

In this study, we aimed to assess the risk of developing VTE in Muslims during fasting days in Ramadan and to suggest a prophylactic protocol against venous thrombosis in Ramadan. We studied 311 persons of both genders in multiple countries, i.e., Egypt, Algeria, Tunisia, Iraq, Lebanon, Syria, and Turkey, with different geographic regions. Overall, 182 (60%) were male participants and 129 (40%) were female participants, with a male-to-female ratio of 3:2. All participants abstained from food and fluids for 30 days (Ramadan month), and the mean fasting duration was 14.02 ± 0.08 hours/day. The age of the participants ranged from 18 to 73 years, with a mean age of 32.08 ± 0.7 years. Overall, 56 (18%) participants were students, 67 (22%) were accountants and managerial workers, 40 (13%) were doctors and healthcare providers, 64 (21%) were engineers and employed in industries, 58 (18.5%) were military and police officers and soldiers, and 25 (7.5%) were housewives.

**Figure 1:**
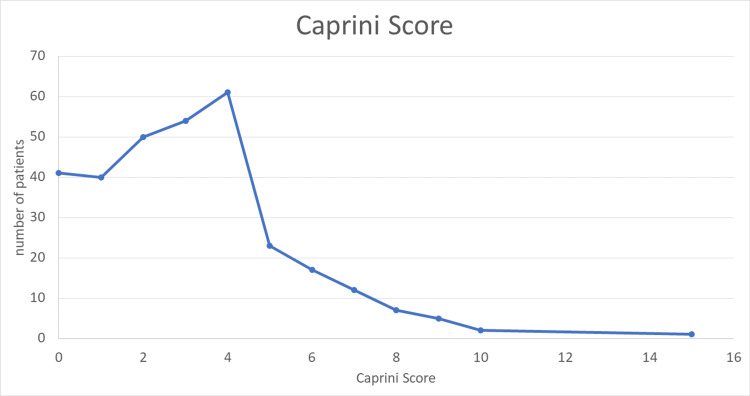
Caprini scores of the study population.

Risk assessment for VTE using the standard Caprini scores for fasting Muslims in the different countries during Ramadan revealed that the mean score was 3.14, with a median of 3.0 and a mode of 4.0, indicating a moderate baseline risk overall. Notably, 47.5% of participants had scores between 3 and 4, classifying them in the moderate-risk category for VTE according to standard Caprini risk interpretation (Figure 1).

**Figure 2:**
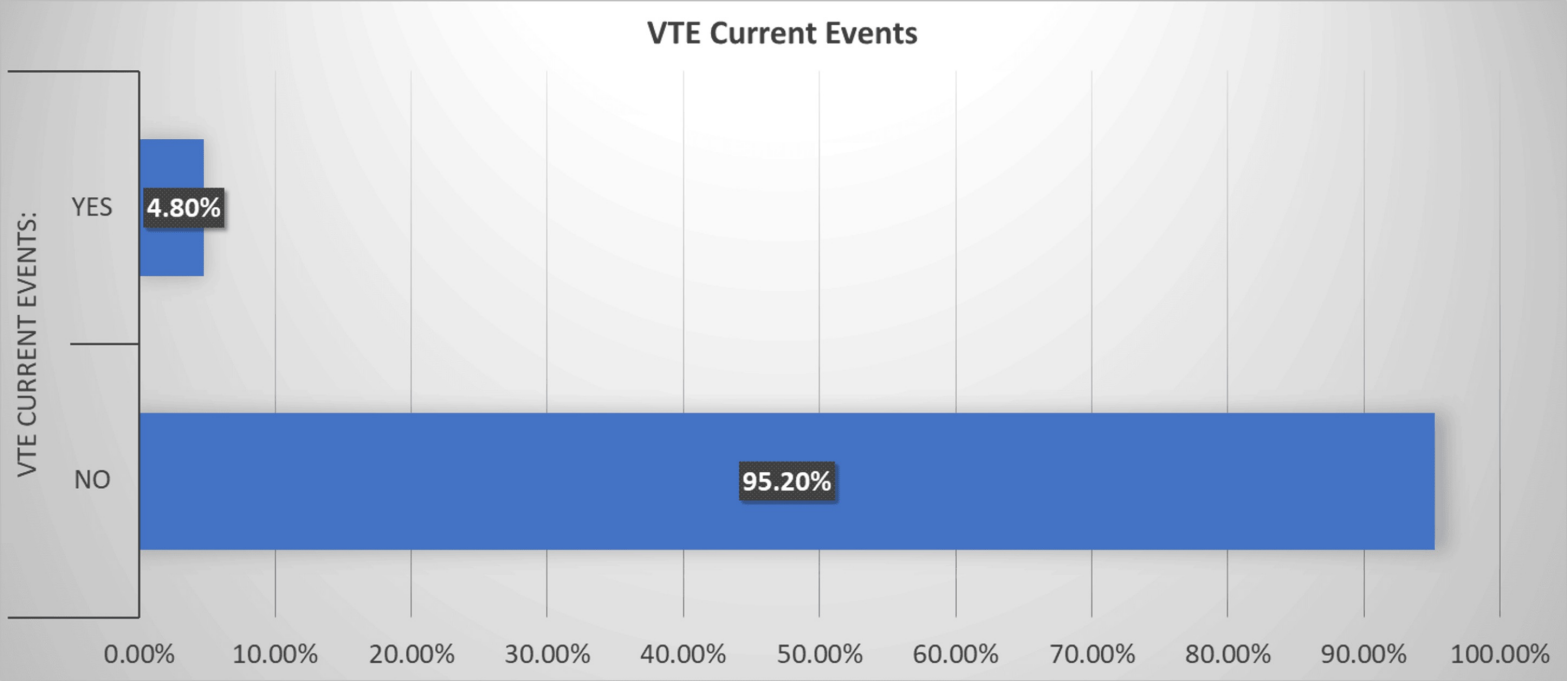
Venous thromboembolism (VTE) current events.

In this study, new-onset events of VTE were observed in 15 (4.8%) participants during the month of Ramadan across various contributing countries. The distribution of VTE subtypes among affected individuals was as follows: superficial thrombophlebitis accounted for four (26.7%), femoropopliteal DVT was the most prevalent at nine (60.0%), followed by isolated calf DVT and iliofemoral DVT, each comprising one (6.7%) case. All thrombotic events occurred during fasting periods lasting between 12 and 15 hours. No fatalities were directly attributable to VTE occurrences. The findings are illustrated in Figure 2.

A significantly higher incidence of VTE was observed among study participants compared to controls who did not develop VTE (13.3% vs. 3.0%; p = 0.034). A strong predictive association was identified: five (30%) participants developed VTE, whereas only nine (2.9%) controls had such an event (p < 0.001). Thrombophilia screening among eight individuals who developed VTE revealed that two were heterozygous for the Factor V Leiden mutation, three tested positive for lupus anticoagulant antibodies, two had anticardiolipin antibodies, and two tested positive for anti-beta-2 glycoprotein I (anti-β2GPI) antibodies. Overall, four (50%) of the eight individuals demonstrated laboratory evidence of thrombophilia. The detailed findings are summarized in Table 3.

**Table 3:** Thrombophilia screening results in patients who developed venous thromboembolism.

Test	Number	Percentage
Antithrombin (previously called antithrombin III)	0	0%
Factor V Leiden	2	25%
Prothrombin gene mutation	0	0%
Protein C	0	0%
Protein S	0	0%
Lupus anticoagulant	3	37.5%
Anti-β-2 glycoprotein 1 antibodies	2	25%
Anticardiolipin antibodies	2	25%

To evaluate the clinical relevance of risk stratification, the Caprini risk scores of participants who developed VTE were compared to those who did not. The mean Caprini score was significantly higher among VTE cases (6.0 ± 2.6) compared to non-VTE participants (3.0 ± 2.2) (p < 0.001; 95% confidence interval = -4.162, -1.845). This reflects a clinically meaningful difference of approximately 3 points.

Upon incorporating additional variables suggested by the risk assessment model into the analysis, it was observed that the duration of daily fasting across all participants ranged uniformly between 12 and 15 hours. Ambient temperature during the fasting period remained consistent among all individuals and did not exceed 30°C. Data on daily physical activity or working hours, as well as total fluid intake per day, were not collected in this study.

An analysis of varicose vein status revealed a strong and statistically significant association with the occurrence of VTE. Overall, 13 (86.7%) VTE patients had varicose veins compared to 112 (38.0%) of non-VTE participants (p < 0.001). Bilateral varicose veins were particularly associated with increased risk, observed in 10 (66.7%) VTE cases versus 94 (31.7%) controls (p = 0.004). Furthermore, advanced CEAP classifications (C2-C4) were significantly more prevalent among VTE patients (p = 0.006). Statistical analysis demonstrated a robust association, with an odds ratio (OR) of 10.64 and a relative risk (RR) of 9.62. The chi-square test yielded a value of 12.12 with a p-value of 0.0005, confirming the strength of the relationship.

In addition, venous reflux was identified in seven (50%) VTE patients compared to 38 (12.7%) controls (p < 0.001). A history of previous varicose vein treatment was reported in five (30%) VTE cases, versus only nine (2.9%) controls (p < 0.001). Table 4 presents further detailed findings.

**Table 4:** Varicose veins condition. CEAP = clinical-etiological-anatomical-pathophysiological; VTE = venous thromboembolism

Varicose veins variables		VTE events		P-value	χ² Value
		No (mean, N)	Yes (mean, N)		
Varicose veins	Absent	181	2	<0.001	12.08
	Present	111	13		
Duration	3.39	7.32 (SD = 13.88)	13.7 (SD = 6.7)	0.010	NA
Side of varicose vein involvement	No Varicose	141	2	0.004	8.97
	Unilateral	29	3		
	Bilateral	79	10		
CEAP category	C0	139	2	0.006	18.17
	C1	31	1		
	C2	47	8		
	C3	24	3		
	C4	5	1		
	C5	4	0		
Venous reflux confirmed on duplex scan	No reflux	179	5	0.001	3.37
	Reflux	26	5		
Previous varicose veins treatment	No	199	7	<0.001	6.49
	Yes	6	3		

**Figure 3:**
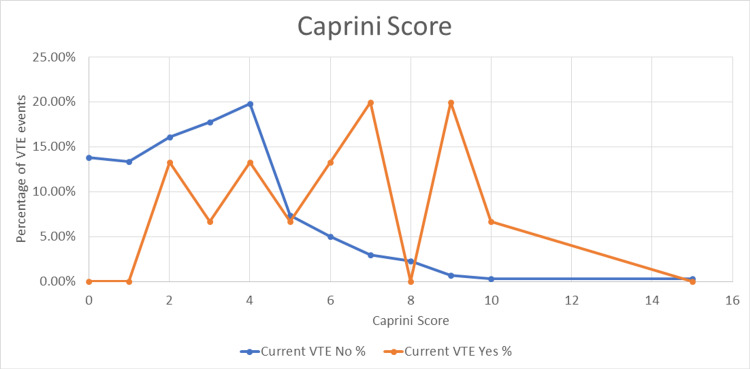
Caprini scores and VTE incidence. VTE = venous thromboembolism

Overall, VTE is uncommon in Ramadan; however, the impact of vigorous dehydration on the coagulation cascade is not yet understood. The combined effect of limited mobility and hereditary clotting disorder could be the key factors. These findings are illustrated in Table 5 and Figure 3.

**Table 5:** Relationship between VTE current events and continuous variables. VTE = venous thromboembolism

Continuous variables	No VTE current events	VTE current events	P-value	95% confidence interval		t-value
	Mean ± SD	Mean ± SD				
Age	44.06 ± 15.892	49.80 ± 15.89	0.173	-14.017	2.531	1.36
Caprini score	3 ± 2.2	6 ± 2.6	<0.001>	-4.162	-1.845	4.39
Duration of varicose veins	13.88 ± 13.7	3.39 ± 7.32	0.067	-21.961	0.981	2.94
Additional score	3 ± 0	3 ± 0	0.196	-.377	0.084	NA
Sum score (Caprini+ additional score)	6.05 ± 2.288	9.20 ± 2.7	<0.001>	-4.353	-1.947	4.44

Cohen’s d effect sizes were calculated to assess the magnitude of differences between VTE and non-VTE groups. For the Caprini score, Cohen’s d was 1.35, indicating a very large effect.

## Discussion

This multicenter, observational study aimed to investigate the potential association between Ramadan fasting in hot climates and the risk of VTE, a topic that remains underexplored despite the known physiological impacts of dehydration on hypercoagulability [2,9]. Our findings revealed a 4.8% incidence rate of VTE among fasting individuals, a notable figure that warrants attention, particularly given the relatively short study duration of 30 days and the sample’s moderate baseline risk, as assessed by the Caprini score. In non-fasting populations with moderate Caprini risk (3-4), typical VTE incidence is estimated at 0.5-1% over a one-month period. Thus, our observed rate is notably elevated. In the study by Elias et al. [10], which included 1,496 patients, with 48% of men, a mean age of 63 ± 18 years, dehydration was suggested to be a potential explanation for the monthly variation in the incidence of VTE [10].

A key finding was the significantly higher mean Caprini score among patients who developed VTE (6.0 ± 2.6) compared to those who did not (3.0 ± 2.2) (p < 0.001). This underscores the utility of the Caprini risk assessment tool in this unique context and suggests that traditional VTE risk stratification remains valid during periods of prolonged fasting. A previous study revealed the risk assessment for VTE of fasting Muslims during Ramadan and suggested a prophylaxis protocol against VTE in Ramadan [11].

Importantly, varicose veins emerged as a strong and independent predictor of VTE in fasting individuals. Among VTE cases, 13 (86.7%) had varicose veins, with bilateral varicosities and higher CEAP classifications (C2-C4), particularly associated with thrombotic events. However, only a very small number of participants in the study were classified as CEAP C5 or C6, limiting the ability to observe associations among this subgroup. Furthermore, venous reflux was significantly more prevalent among those who developed VTE (50% vs. 12.7%, p < 0.001), as was a history of previous VTE or varicose vein treatment. These findings suggest that fasting-related dehydration may act synergistically with pre-existing venous pathology to precipitate thrombotic events. A 2022 study demonstrated that genetically predicted varicose veins may have a causal effect on DVT and may be one of the mediators of obesity and taller height that predispose to DVT [12].

While the fasting duration (12-15 hours) and ambient temperature (<30°C) were uniform across participants, it is notable that even under these relatively mild conditions, thrombotic events occurred. This implies that risk factors intrinsic to the individual (e.g., varicose veins, prior VTE, thrombophilia) may be more critical than external environmental factors in determining VTE risk during Ramadan. It also raises the concern that more extreme fasting durations or higher temperatures could further exacerbate the risk. Another study by Manfredini et al. [13] demonstrated the existence of a seasonal and monthly variability in the occurrence of VTE. The incidence of VTE is significantly higher in the winter, in particular, in the month of January, with an absolute increase of 12% and 20%, respectively [13].

Thrombophilia and a prior history of VTE were also significantly associated with incident thrombotic events, aligning with established literature. Dicks et al. [14] examined the role of thrombophilia evaluation in VTE patients, including the indications and timing of performing such an evaluation [14]. Notably, two (13.3%) VTE patients tested positive for thrombophilia versus nine (3.0%) in the non-VTE group (p = 0.034), and five (30%) had a prior VTE history (p < 0.001). These findings highlight the need for targeted risk mitigation strategies in high-risk populations during Ramadan, such as adjusted fluid intake strategies, activity scheduling, or even pharmacologic prophylaxis for those with prior VTE or severe varicosities.

Despite the robust associations found, the study has limitations. Key variables such as exact fluid intake and physical activity levels were not quantified, limiting the analysis of their potential mediating effects. The study also lacked longitudinal follow-up, preventing assessment of post-Ramadan complications or recurrence rates. Moreover, the generalizability of the findings is confined to similar climate zones and fasting durations. Smoking status was not recorded, which may represent confounding variables affecting thrombosis risk. Future studies should consider incorporating these factors. On the other hand, participant enrollment was site-based and not randomized. This introduces the potential for selection and referral bias, especially in centers with higher clinical volume or vascular specialization. The internal comparisons were made between VTE and non-VTE participants within the same cohort, lacking an external control group.

## Conclusions

This study identifies an association between elevated Caprini scores and increased VTE incidence during Ramadan fasting, even under moderate durations and temperatures, which may increase the risk of venous thromboembolism in individuals with predisposing factors such as varicose veins, a history of thrombosis, or thrombophilia. The Caprini risk assessment model, when supplemented with fasting-specific considerations, proved useful in identifying individuals at elevated risk. To ensure safe fasting during Ramadan, healthcare providers should consider proactive risk assessment and education for at-risk populations. Strategies may include encouraging adequate hydration during non-fasting hours, promoting mobility, and considering prophylactic measures when warranted. While universal thrombophilia screening is not practical, selective screening may be helpful in individuals with known risk factors, family history, or prior events. Future research should further explore preventive approaches and the impact of environmental and behavioral variables on thrombosis risk in fasting populations.
